# Carbon budget of different forests in China estimated by an individual-based model and remote sensing

**DOI:** 10.1371/journal.pone.0285790

**Published:** 2023-10-09

**Authors:** Junfang Zhao, Jinlong Ai, Yujie Zhu, Ruixi Huang, Huiwen Peng, Hongfei Xie

**Affiliations:** 1 State Key Laboratory of Severe Weather, Chinese Academy of Meteorological Sciences, Beijing, 10081, China; 2 School of Modern Agriculture, Yiyang Vocational & Technical College, Hunan, 413049, China; 3 CMA Institute for Development and Programme Design (CMAIDP), Beijing, 10081, China; Tennessee State University, UNITED STATES

## Abstract

Forests play a key role in the regional or global carbon cycle. Determining the forest carbon budget is of great significance for estimating regional carbon budgets and formulating forest management policies to cope with climate change. However, the carbon budget of Chinese different forests and their relative contributions are not completely clear so far. We evaluated the carbon budget of different forests from 1981 to 2020 in China through combining model with remote sensing observation. In addition, we also determined the relative contribution of carbon budget of each forest type to all forests in China. Eight forest types were studied: evergreen coniferous forest (ECF), deciduous coniferous forest (DCF), coniferous and broad-leaved mixed forest (CBF), deciduous broad-leaved forest (DBF), evergreen broad-leaved forest (EBF), evergreen deciduous broad-leaved mixed forest (EDBF), seasonal rain forest (SRF), and rain forest (RF). The results indicated that the Chinese forests were mainly carbon sink from 1981 to 2020, particularly the annual average carbon budget of forest from 2011 to 2020 was 0.191 PgC·a^-1^. Spatially, the forests’ carbon budget demonstrated obvious regional differences, gradually decreasing from Southeast China to Northwest China. The relative contributions of carbon budget in different forests to all forests in China were different. During 2011–2020, the ECF forests contributed the most carbon budget (34.45%), followed by DBF forests (25.89%), EBF forests (24.82%), EDBF forests (13.10%), RF forests (2.23%), SRF forests (3.14%) and CBF forests (1.14%). However, the DCF forests were found mainly as carbon source. These results contribute to our understanding of regional carbon budget of forests.

## Introduction

Human activities lead to the increased carbon dioxide concentration in the atmosphere, and changed climatic conditions [1]. Climate change, in turn, can change the carbon cycle, vegetation productivity and species distribution of ecosystems. The ecosystem carbon cycles include several processes and components: total amount of organic carbon fixed by green plants through photosynthesis (gross primary productivity, GPP), released carbon by autotrophic respiration (Ra) and soil heterotrophic respiration (Rh) [2]. The value of GPP minus Ra is net primary productivity (NPP). The value of NPP minus Rh is net ecosystem productivity (NEP), namely the carbon budget [3]. A clear regional NEP will be an important supplement to reveal the global carbon cycle mechanisms and reduce the uncertainties in global NEPs [4–6].

As an important part of terrestrial ecosystem, forest ecosystem has enormous carbon pool and biodiversity [7]. Forest ecosystems can provide human livelihood, produce bioenergy, fix carbon dioxide and slow down climatic change [8]. China has rich forest resources and diverse forest types, with obvious zonal distribution characteristics. According to the results from Chinese Ministry of Forestry [9], the global largest plantation is in China. Moreover, China has two-thirds young forests with great carbon sequestration potentials [10]. In order to achieve the carbon neutrality goal by 2060, China is required to vigorously promote carbon fixation and emission reduction. Consequently, quantitatively evaluating the forests’ NEP at different time and space scales is very crucial for formulating effective management strategies and providing important references for the realization of carbon neutrality in China.

The evaluation technologies of forests’ NEP are continuously improved over the past few decades. At present, these methods mainly involve the positioning observation, carbon flux observation and model simulation [10]. Generally speaking, in large scales, the traditional methods of positioning observation and carbon flux observation are easily influenced by some elements such as observation, survey, measurement, funds, and so on. That is because the changes in carbon cycle of forests not only stride across seasons, years and decades, but also vary spatially with regional climate, environments, and vegetation types [11]. Owing to the complex mechanisms of carbon cycle process, the model simulation has become an irreplaceable mean for estimating forests’ NEP in large spatial scale and long-term range [12].

More researchers have shifted their interests from individual ecosystems to larger zones and even to the global scale. So far, many studies have applied ecosystem process models to assess the large-scale forest carbon cycle [13–20]. For example, Caddeo et al. [15] applied the CENTURY model to estimate the current and future soil organic carbon stocks of the whole forests in Italy at 2005 and at 2095 under climate change. Derroire et al. [17] developed a temporally-explicit and territory scale model of carbon balance and recommend a mixed-strategy for improving long-term carbon balance and reducing short-term emissions by combining selective logging in natural forests and plantations in French Guiana. Then, Gong et al. [19] evaluated the carbon fluxes from contemporary forest disturbances in North Carolina based on a Grid-based Carbon Accounting (GCA) model. Kimberley et al. [20] used the Coarse Woody Debris (CWD) carbon stock model to compare the measured and modelled changes in CWD carbon stocks in New Zealand’s natural forest. The above researches further show the model method has become very feasible and can improve the simulation results of large-scale forest carbon cycles. However, so far, some uncertainties are still existed in estimating forests’ NEP in China. There are few national level studies on the NEPs and their relative contributions of different forests to all forests in China. Clarifying regional NEP of different forest types is crucial for sustainable development in China.

Here, we investigated the NEP of different forest types in China through the individual-based model and remote sensing products. We also explored the NEP of Chinese different forests and their relative contributions with long time series data. Our study was designed to provide scientific supports for reasonably evaluating on regional NEP of different forest types.

## Materials and methods

### Main distributions of Chinese forests

In general, the forests on the earth can be divided into coniferous forest, coniferous broad-leaved mixed forest, deciduous broad-leaved forest and evergreen broad-leaved forest. These forests are distributed in different regions according to different climatic factors. However, due to the combination difference of water and heat, the forests in various regions show great differences. With vast territory and complex natural climatic conditions, China has rich forest resources and diverse forest types, with obvious zonal distribution characteristics. Chinese forests according to their generic characteristics and habitats are divided into eight types: (1) evergreen coniferous forest (ECF), (2) deciduous coniferous forest (DCF), (3) coniferous and broad-leaved mixed forest (CBF), (4) deciduous broad-leaved forest (DBF), (5) evergreen broad-leaved forest (EBF), (6) evergreen deciduous broad-leaved mixed forest (EDBF), (7) seasonal rain forest (SRF) and (8) rain forest (RF).

Coniferous forests are widely distributed in China. Northern coniferous forest and subalpine coniferous forest, which are high latitude horizontal zonal vegetation and low latitude subalpine vegetation types respectively, differing greatly in distribution and geographical environment. Generally speaking, they all belong to the sub cold zone type, with similar appearance, composition and structure. The coniferous forests in warm temperate are mainly in Liaodong Peninsula and North China. There are many types of subtropical coniferous forests. Tropical coniferous forest has few tree species, which are scattered and not forested. The coniferous broad-leaved mixed forests include Korean pine broad-leaved mixed forests and Hemlock broad-leaved mixed forest. Pinus koraiensis broad-leaved mixed forests are zonal types in temperate regions of China, mainly distributed in the Changbai Mountain and Xiaoxing’an Mountain in Northeast China. Hemlock broad-leaved mixed forests are mainly distributed in the mountainous subtropical areas of China. Deciduous broad-leaved forests are widely distributed in temperate zone, warm temperate zone and subtropical zone. Evergreen broad-leaved forests are zonal types in humid subtropical forest area of China. The monsoon rain forests are the representative vegetations of monsoon tropics in China, and most of them are distributed in the arid hilly tableland, basin and valley areas. Rain forest is mainly distributed in the mountains above 500m-700m above sea level in tropical areas of China.

### Brief introduction of FORCCHN model

The FORCCHN (FORest ecosystem Carbon budget model for CHiNa) model was applied in this study, and was used to estimate forests’ NEP under changing environments, obviously improving the evaluation abilities of forests’ NEP [21]. This model with spatial resolution of 10 km×10 km is based on individual tree. It consists of five modules: initialization submodule, carbon balance submodule, ecoclimatic submodule, soil carbon and nitrogen budget submodule, and tree growth submodule [21]. The FORCCHN model calculates the NEP of each tree on a certain patch one by one, and obtains the NEP of the ecosystem per unit area by summing and coupling the soil NEP calculated by the soil carbon cycle model.

**Fig 1:**
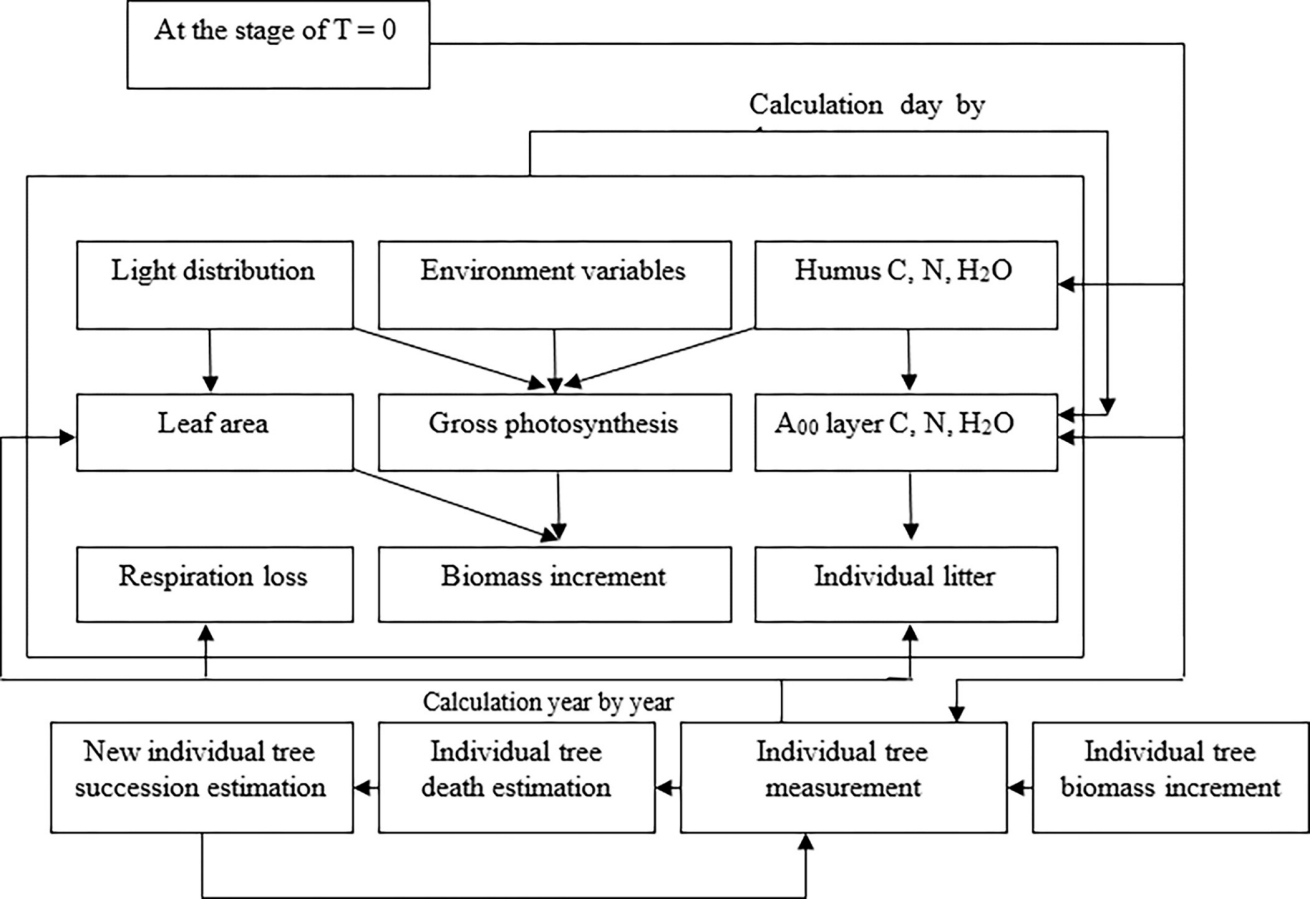
Primary processes and flow chart of the FORCCHN model.

The FORCCHN model runs in daily or annual process step. For daily step, the photosynthesis, continuous respiration, litter, and nitrogen absorption of each tree are computed daily. Soil moisture, soil organic matter decomposition and nitrogen mineralization are computed daily. Furthermore, in any time of day, the rate of maintenance respiration in each tree, litter of leaf and fine-root, soil organic matter decomposition and nitrogen mineralization are assumed to be the same. For annual step, the changes in assimilate, tree height, diameter at breast height, and litter are be calculated. Specifically, the annual structure growth carbon and the fruit litter of each tree are computed through accumulated NEP every day. Then, the tree growths in height and diameter are be calculated every year. Finally, increased and stored carbon are assigned to each component. The main processes in FORCCHN model are displayed in Fig 1. The input, output and main characteristics of the FORCCHN model are shown in Table 1.

**Table 1:** Main characteristics of the FORCCHN model.

Characteristic	Detailed description
Initial conditions	Field water holding capacity, soil carbon storage, soil nitrogen storage, and remote sensing LAI data.
Boundary variables	Daily maximum temperature, minimum temperature, average temperature, precipitation, relative air humidity, total radiation, average wind speed, average air pressure, and atmospheric CO_2_ concentration.
Material balance scheme	Complete carbon balance, and nitrogen and water in the atmospheric-soil-forest ecosystem
Time step and scheme	Daily carbon and nitrogen uptake, litter flux and respiration flux per tree; Daily soil carbon, nitrogen, and water are imported and exported; Daily forest carbon and nitrogen uptake and litter flux in patches; Calculations of carbon accumulation per tree, flower, and fruit litter flux, and tree DBH; and growth, tree height growth, and subbranch height growth year by year.
Carbon and nitrogen budget module per tree per day	Considering total photosynthesis, maintenance respiration, growth respiration, distribution, and the litter of photosynthate, the photosynthate buffer pool scheme was adopted to enhance resistance to extreme climatic conditions.
Daily soil carbon and nitrogen budget module	An improved CENTURY model suitable for forest soils is adopted so that the decomposition and respiration of forest soils can be temporarily considered as valid in the absence of validation data.
Annual tree growth	Calculations of annual photosynthate distribution, flower and fruit litter, tree DBH, tree height, under-branch height, and potential maximum leaf volume in the buffer pool.

Main formulas for calculating NEP are as follows:

(1) Gross primary productivity:
$${GPP}_{d}=min({GPP}_{m}\times F_{c}\times F_{w}\times F_{t},\alpha \times N_{s})$$ (1)
  where *GPP*_*d*_ is the daily gross primary productivity (kgC·m^-2^·d^-1^); *GPP*_*m*_ is the daily maximum gross primary productivity (kgC·m^-2^·d^-1^); *F*_*c*_ is the effect of atmospheric CO_2_ concentration on gross primary productivity; *F*_*w*_ is the effect of soil water on gross primary productivity; *F*_*t*_ is the effect of air temperature on gross primary productivity; *a*×*N*_*s*_ is the effect of soil available nitrogen on gross primary productivity; *a* is the carbon nitrogen ratio parameter of assimilation, with a value of 150; *N*_*s*_ is the soil available nitrogen (kgN·m^-2^·d^-1^).
$${\mathrm{GPP}}_{m}=\frac{2\times A_{m}\times D}{K}ln\left[\frac{1+\sqrt{1+K\times S\times PAR/A_{m}}}{1+\sqrt{1+K\times S\times PAR\times \exp(-K\times LAI)/A_{m}}}\right]$$ (2)
  where *GPP*_*m*_ is the daily maximum gross primary productivity (kgC·m^-2^·d^-1^); *A*_*m*_ is the possible maximal photosynthesis of leaves (kgC·m^-2^·h^-1^); *D* is the sunshine hours (h); *K* is the extinction coefficient; *S* is the initial slope of light intensity and photosynthesis; *PAR* is the canopy photosynthetic active radiation at noon (W·m^-2^); *LAI* is theleaf area index.
$$F_{c}=1+\frac{C_{t}-C_{0}}{C_{t}+2\times C_{0}}$$ (3)
  where *F*_*c*_ is the effect of atmospheric CO_2_ concentration on gross primary productivity; *C*_*t*_ is the average CO_2_ concentration of the year (ppm); *C*_0_ is the reference CO_2_ concentration of the year (ppm).
$$F_{w}={\left\{\frac{min[1,W_{s}/W_{f}+max(R_{h}-0.5,0.1)]}{W}\right\}}^{1/2}$$ (4)
  where *F*_*w*_ is the effect of soil water on gross primary productivity; *W*_*s*_ is the soil water content (cm); *W*_*f*_ is the soil field capacity (cm); *R*_*h*_ is the air relative humidity; *W* is the drought tolerance of trees.
$$F_{t}={\left(\frac{T_{max}-T}{T_{max}-T_{opt}}\right)}^{\frac{T_{max}-T_{opt}}{T_{max}-T_{min}}}\times {\left(\frac{T-T_{min}}{T_{opt}-T_{min}}\right)}^{\frac{T-T_{min}}{T_{opt}-T_{min}}}$$ (5)
  where *F*_*t*_ is the effect of air temperature on gross primary productivity; *T*_max_ is the maximum temperature for photosynthesis (°C); *T* is the average temperature of the day (°C); *T*_opt_ is the optimum photosynthetic temperature (°C); *T*_min_ is the minimum temperature at which photosynthesis (°C).
(2) Annual net primary productivity:
$$NPP=\sum_{d=1}^{365}\left({GPP}_{d}-R_{m}-R_{g}\right)$$ (6)
  where *GPP*_*d*_ is the daily gross primary productivity (kgC·m^-2^·d^-1^); *R*_*m*_ is the daily maintenance respiration (kgC·m^-2^·d^-1^); *R*_*g*_ is the daily growth respiration (kgC·m^-2^·d^-1^).
$$R_{m}=\frac{1}{24}[D\times e^{0.069315\times (T_{d}-15)-0.009\times {\left(T_{d}-15\right)}^{2}}+(24-D)\times e^{0.069315\times (T_{n}-15)-0.009\times {\left(T_{n}-15\right)}^{2}}]\times R_{k}\times C_{k}$$ (7)
  where *R*_*m*_ is the daily maintenance respiration (kgC·m^-2^·d^-1^); *D* is the sunshine hours (h); *T*_*d*_ is the daytime average temperature(°C); *T*_*n*_ is the average temperature at night (°C); *R*_*k*_ is the relative respiration rate of leaves, trees and roots at 15°C (d^-1^); *C*_*k*_ is the amount of corresponding carbon pool. When *k* represents leaves and fine roots, *C*_*K*_ is the amount of leaves and fine roots; and when *k* represents branches, stems and roots, *C*_*K*_ is the amount of sapwood (kgC).
$$R_{g}=r_{g}\times ({GPP}_{d}-R_{m})$$ (8)
  where *R*_*g*_ is the daily growth respiration(kgC·m^-2^·d^-1^); *r*_*g*_ is the growth respiration coefficient, with the valued of 0.25; *GPP*_*d*_ is the daily gross primary productivity (kgC·m^-2^·d^-1^); *R*_*m*_ is the the daily maintenance respiration (kgC·m^-2^·d^-1^).
(3) Annual soil heterotrophic respiration:
$$R_{s}=\sum_{u=1}^{10}P_{u}\times S_{u}\times G_{T}\times G_{W}\times e^{-b\times L_{s}}\times C_{u}$$ (9)
  where *R*_*s*_ represents the annual soil heterotrophic respiration (kgC·m^-2^·a^-1^); *P*_*u*_ is the proportion of respiration in the *u*th carbon pool; *S*_*u*_ is the reference relative decomposition rate of the *u*th carbon pool; *G*_*t*_ is the influence coefficient of temperature on decomposition process; *G*_*w*_ is the influence coefficient of water on decomposition process; *b* is a constant, with a value of 5.0; *L*_*s*_ represents the lignin content of structural litter pool; *C*_*u*_ is the difference between soil carbon pool and lignin in the *u*th carbon pool (kgC·m^-2^); *u* is the *u*th soil carbon pool.
$$G_{t}=e^{\frac{3.36\times (T_{s}-40)}{T_{s}+31.79}}$$ (10)
  where *G*_*t*_ is the influence coefficient of temperature on decomposition process; *T*_*s*_ is the soil temperature(°C).
$$G_{W}=1-(\frac{W_{s}}{e\times W_{f}}-1{)}^{2}$$ (11)
  where *G*_*w*_ is the influence coefficient of water on decomposition process; *W*_*s*_ is the soil water content (cm); *e* is a constant, with a value of 0.6; *W*_*f*_ is the Field capacity (cm).

The reference relative decomposition rates of soil carbon pool in this study are shown in the Table 2.

**Table 2:** Reference relative decomposition rates of soil carbon pool (d^-1^).

*S*_1_	*S*_2_	*S*_3_	*S*_4_	*S*_5_	*S*_6_	*S*_7_	*S*_8_	*S*_9_	*S*_10_
0.021	0.1	0.027	0.13	0.01	0.002	0.002	0.042	0.001	3.5×10^−5^

Note: *S*_1_ represented the above-ground metabolic litter pool; *S*_2_ represented the above-ground structural litter pool; *S*_3_ represented the below-ground metabolic litter pool; *S*_4_ represented the below-ground structural litter pool; *S*_5_ represented the fine woody litter pool; *S*_6_ represented the coarse woody litter pool; *S*_7_ represented the below-ground coarse litter pool; *S*_8_ represented the active soil organic matter pool; *S*_9_ represented the slow soil organic matter pool; *S*_10_ represented the resistant soil organic matter pool.

(4) Annual carbon budget:

$$NEP=NPP-R_{s}$$ (12)

where *NEP* represents the annual carbon budget of tree (kgC·m^-2^·a^-1^); *NPP* represents the annual net primary productivity (kgC·m^-2^·a^-1^); *R*_*s*_ represents the annual soil heterotrophic respiration (kgC·m^-2^·a^-1^).

Yan and Zhao [21] and Zhao et al. [19] previously provided descriptions on the building strategy, structure, and parameters of FORCCHN model. Here, we focused on verifying FORCCHN model and investigating the NEP dynamics of different forest types and their contributions to forest’s total NEP in China.

### Data

#### Meteorological data

We collected 2423 meteorological station data from 1981 to 2020 from the National Meteorological Information Centre in China. The details of these daily meteorological data were mainly average temperature (°C), minimum temperature (°C), maximum temperature (°C), precipitation(mm), sunshine hours (h), longitude (°) and latitude (°). These daily station data were interpolated into grid data (10 km×10 km) according to the reverse distance and nearest field methods, in order to be consistent with the resolution of the FORCCHN model [21]. According to the principle of reverse distance, the influence of a measured value on the interpolation target point decreased with the increase of the spatial distance between the target point and the measured value. According to the nearest field principle, a given observation value was inversely proportional to the spatial density of observation values in its surrounding action area (the interpolation influence area) and its local space. In other words, the higher the density of observation values in a spatial area, the smaller the effective influence area of each measurement value. Finally, all the meteorological elements in China used in this study (10 km×10 km) were acquired, including daily temperature, precipitation and sunshine hours. In order to calculate the solar radiation every day, the algorithm estimating the solar radiation attenuation by computing the atmosphere transparency was used [22]. Moreover, this algorithm also considered the effects of snow cover and snowmelt on solar radiation in the winter and in the spring.

#### Soil data

We collected 1:14 million soil quality maps provided by the Institute of Soil Science, Chinese Academy of Sciences. These data mainly consist of soil organic matter parameters and soil physical parameters. The parameters of soil organic matter are soil carbon pool (kgC·m^-2^) and soil nitrogen pool (kgN·m^-2^). The parameters of soil physics are soil field capacity (cm), sand content (%), silt content (%), clay content (%), bulk density (KgC·m^-3^) and soil water content (cm) at wilting point. In this study, the soil data in 1981 were interpolated into grid data (10 km×10 km), in order to be consistent with the resolution of the FORCCHN model.

#### Vegetation data

The parameters of forests are composed of forest types, coverage rate (ratio of forest area to total land area), and maximum LAI. The vegetation classification of China was from the 1:1 million vegetation map of China provided by Institute of Geographic Sciences and Natural Resources Research, Chinese Academy Sciences (https://www.resdc.cn/data.aspx?DATAID=122). At the same time, the coverage rates of the forest grids were also referred to the existing research result [23]. In addition, the LAI data in 1981 used in this study were obtained through remote sense image inversion method [24]. Considering that it was impossible to directly obtain the regional forest LAI, the forest LAI at initial field was calculated through the remote sensing model based on the normalized difference vegetation index (NDVI) dataset. NDVI data were from NASA’s Pathfinder AVHRR NDVI land data sets, with the 16km×16 km spatial resolution, compressed text record format, and Goode projection coordinate system. Part parameters in different forest types are showed in Table 3.

**Table 3:** Part parameters in different forest types.

Forest types	NDVI_max_	NDVI_min_	LAI_v_
Evergreen coniferous forest	0.687	0.033	8.0
Deciduous coniferous forest	0.687	0.033	8.0
Deciduous broad-leaved forest	0.687	0.033	7.0
Coniferous and broad-leaved mixed forest	0.687	0.033	7.5

### Running the FORCCHN model

In this study, all data in the year of 1981 were run in FORCCHN model (10 km×10 km) several times until the initialization equilibrium (spin-up) state was reached in order to remove the effects of initial value of hypothetical variable in ecosystem on dynamic simulation. After that, we used all the daily data during 1981–2020 to drive the FORCCHN model for simulation. We mainly focused on the dynamics in NEP of different forest types during 2011–2020.

### Data processing and analysis

We used ArcGIS10.6, SPSS20, and Microsoft Excel 2019 for handing data, statistical analysis and visualization mapping in this study.

### Model accuracy evaluation

The model validation is to compare the simulation results with the actual observation qualitatively and quantitatively. The simulation results of FORCCHN model had been tested by actual observations from 690 sample plots based on forest inventory [21], it was considered to be valid.

### Model parameter acquisition

Generally, model parameters can be obtained by three different means: (a) using control experiments to measure the parameters of simple physical and chemical processes; (b) the various composite parameters of physical characteristics can be fitted by replacing the observed variable values into the mode; (c) the parameters can be obtained from relevant literatures; (d) there might be a few parameters that can only be fitted by multiple simulations. Among the above approaches, the method (b) and the method (c) were used in this study [21]. Because in ecological research, there are little possibilities of controlling experiments.

## Results

### Applicability of FORCCHN model for Chinese forests at different scales

**Fig 2:**
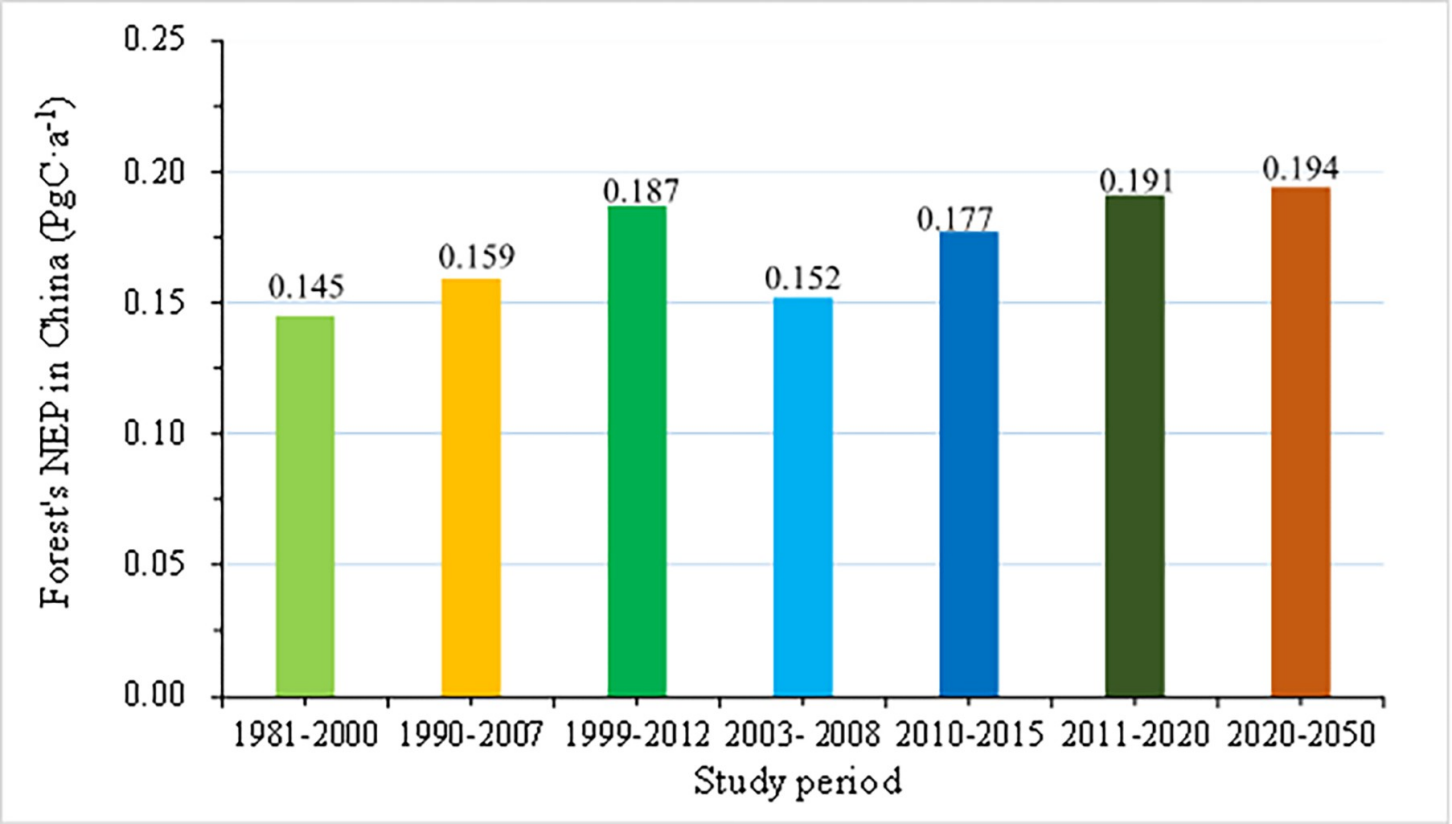
Comparison between NEP simulated by FORCECHN model and other research results on the countrywide scale.

Here, we forced FORCCHN model and compared the simulated total NEP of Chinese forests with previous simulations and observations at the national level (Fig 2). These results from the forest inventory data and the model simulation all showed the Chinese forests in the past few decades were obvious carbon sink. Average annual NEP of forests was 0.115 PgC·a^-1^~0.145 PgC·a^-1^ during 1981–2000 [25], 0.159 PgC·a^-1^ during 1990–2007 [13], 0.187 PgC·a^-1^ during 1999–2012 [26], 0.152 PgC·a^-1^ during 2003–2008 [27], 0.177 PgC·a^-1^ during 2010–2015 [28] and 0.194 PgC·a^-1^ during 2020–2050 with the steadily increasing quality and rapidly growing of young forests [27]. Our results simulated by FORCCHN model showed the Chinese forests from 2011 to 2020 were also carbon sink, with 0.191 PgC·a^-1^ annual average NEP. Our results were consistent with existing research conclusions [13,25–27].

**Fig 3:**
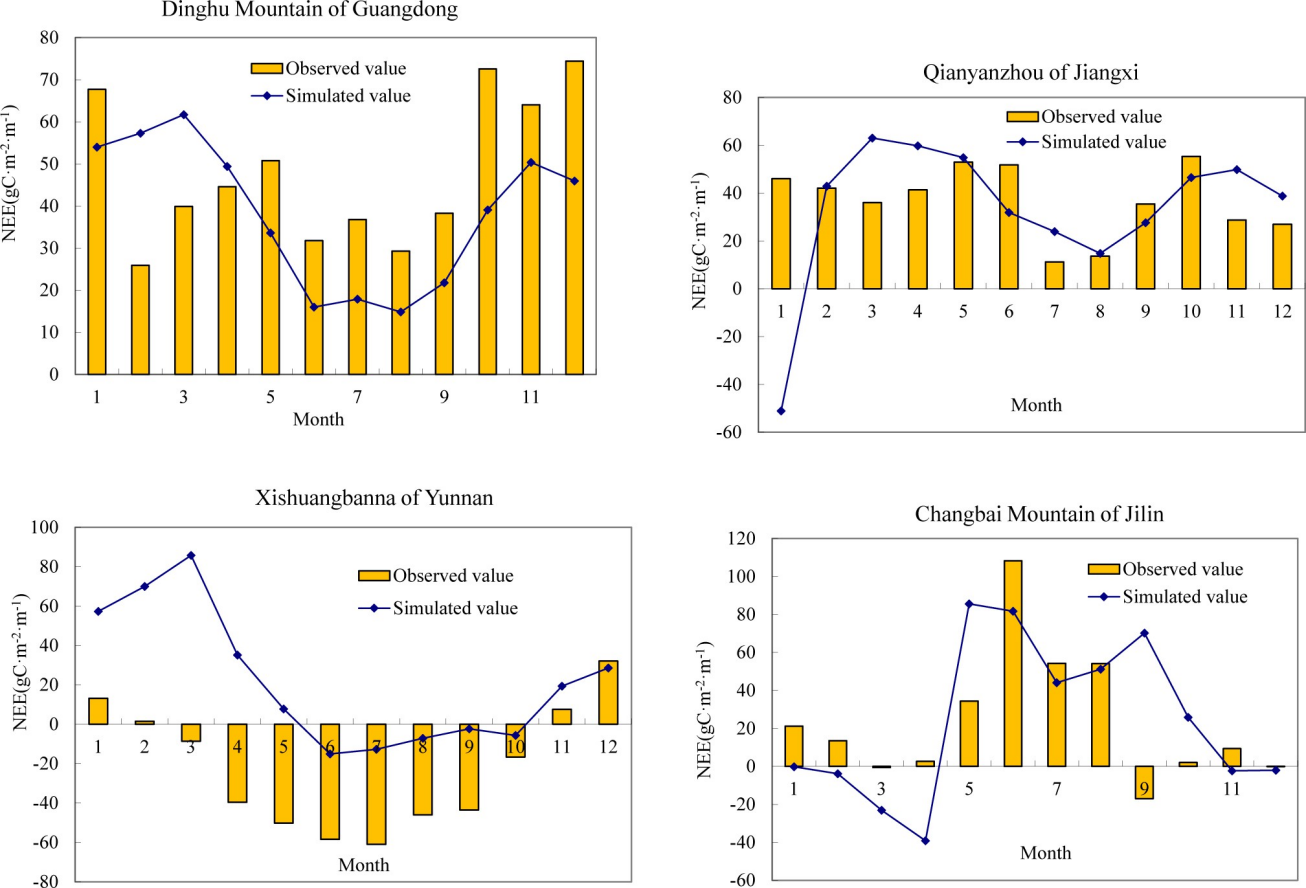
Comparison of monthly variations between simulated NEP with observed NEE in four ecosystem flux stations in 2003.

Net ecosystem carbon exchange (NEE) refers to the CO_2_ flux between land and atmosphere, which is consistent with the observed CO_2_ flux of ecosystems. Generally, NEE and NEP are equal. Therefore, this study compared the simulated NEP with the observed NEE of Chinese forests. The NEE and NEP in 2003 were selected for comparative analysis, mainly considering that the data in 2003 were relatively complete. The monthly simulated NEP in 2003 and observed NEE from the four ecosystem flux stations in Dinghu Mountain of Guangdong, Xishuangbanna of Yunnan, Qianyanzhou of Jiangxi and Changbai Mountain of Jilin were compared and analyzed. Fig 3 shows the comparison between simulated and observed values of NEP in forest ecosystem at station scales. It was found that except for individual months, the results of this study could generally reflect the monthly change characteristics of forests’ NEP. It can be seen from the above analysis that the FORCCHN model could well simulate the spatiotemporal dynamics of NEP in Chinese forests.

### Distribution pattern of carbon budget of Chinese forests

**Fig 4:**
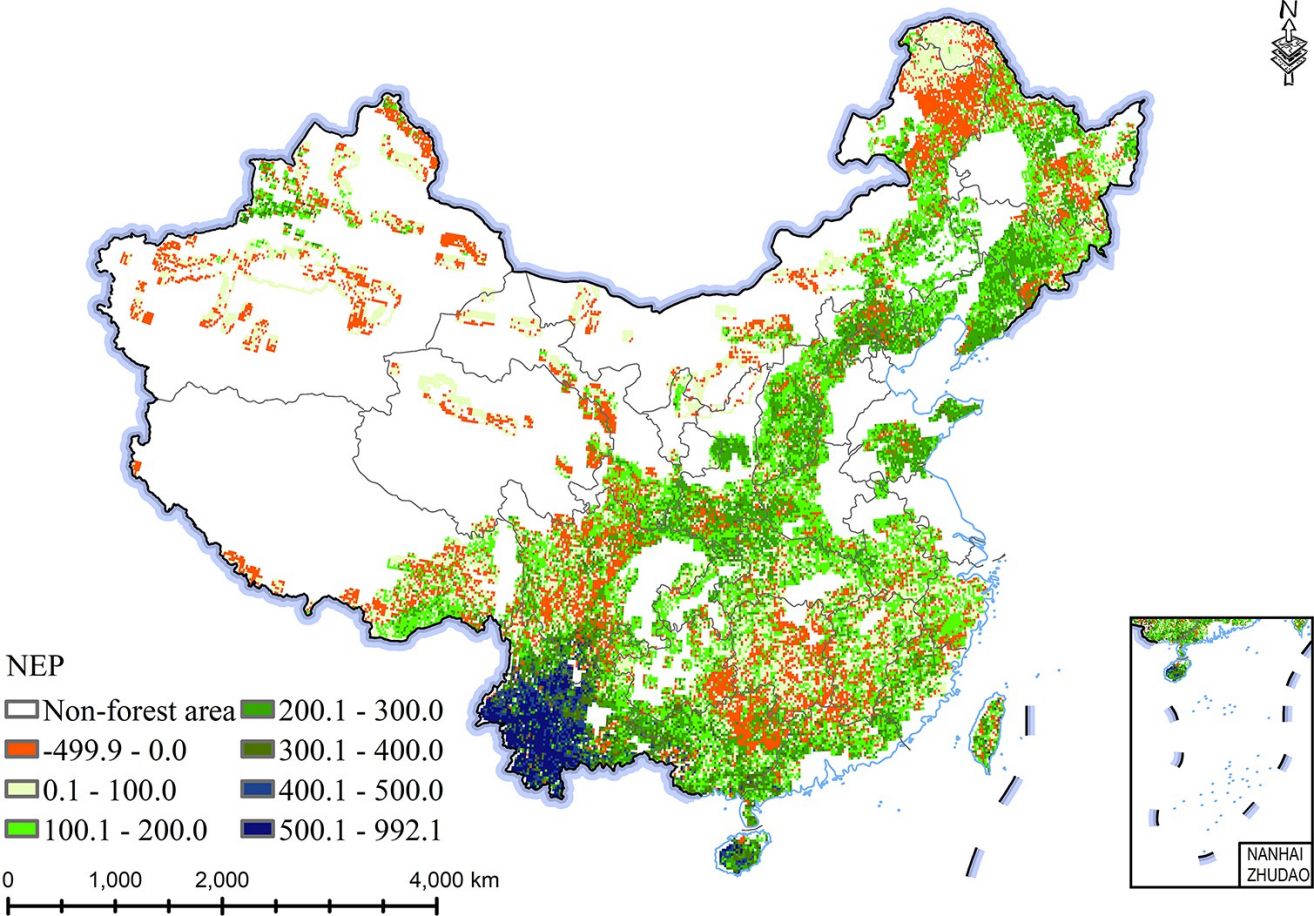
Average distribution of NEP of forests in China from 2011 to 2020 (gC·m^-2^·a^-1^).

Over the past 40 years, Chinese forests were mainly carbon sinks. The spatial distributions of forests’ NEP demonstrated distinct spatial differences, with gradual decreasing from Southeast China to Northwest China. In particular, the higher NEP of forest during 2011–2020 appeared in Southwest China, followed in Southeast China and Northeast China, and lower higher NEP of forest per unit area appeared in Northwest China (Fig 4). The forest in the southwestern region had the highest NEP and the strongest carbon sink capacity. In particular, from 2011 to 2020, the NEP of forest in Yunnan Province was 500.1 gC·m^-2^·a^-1^ ~ 992.1gC·m^-2^·a^-1^. The forests’ NEP in Southeast China was higher, and the NEP in most areas ranged from 200 gC·m^2^·a^-1^ to 400 gC·m^2^·a^-1^. However, some forests in the Northeast China and Northwest China played the roles of carbon source. The forests’ NEP per unit area in some scattered areas of Inner Mongolia and Heilongjiang Province was less than 0. The most carbon released into the atmosphere was mostly found in the northern Heilongjiang Province.

### Carbon budget of different forests and their contribution to Chinese forests

**Fig 5:**
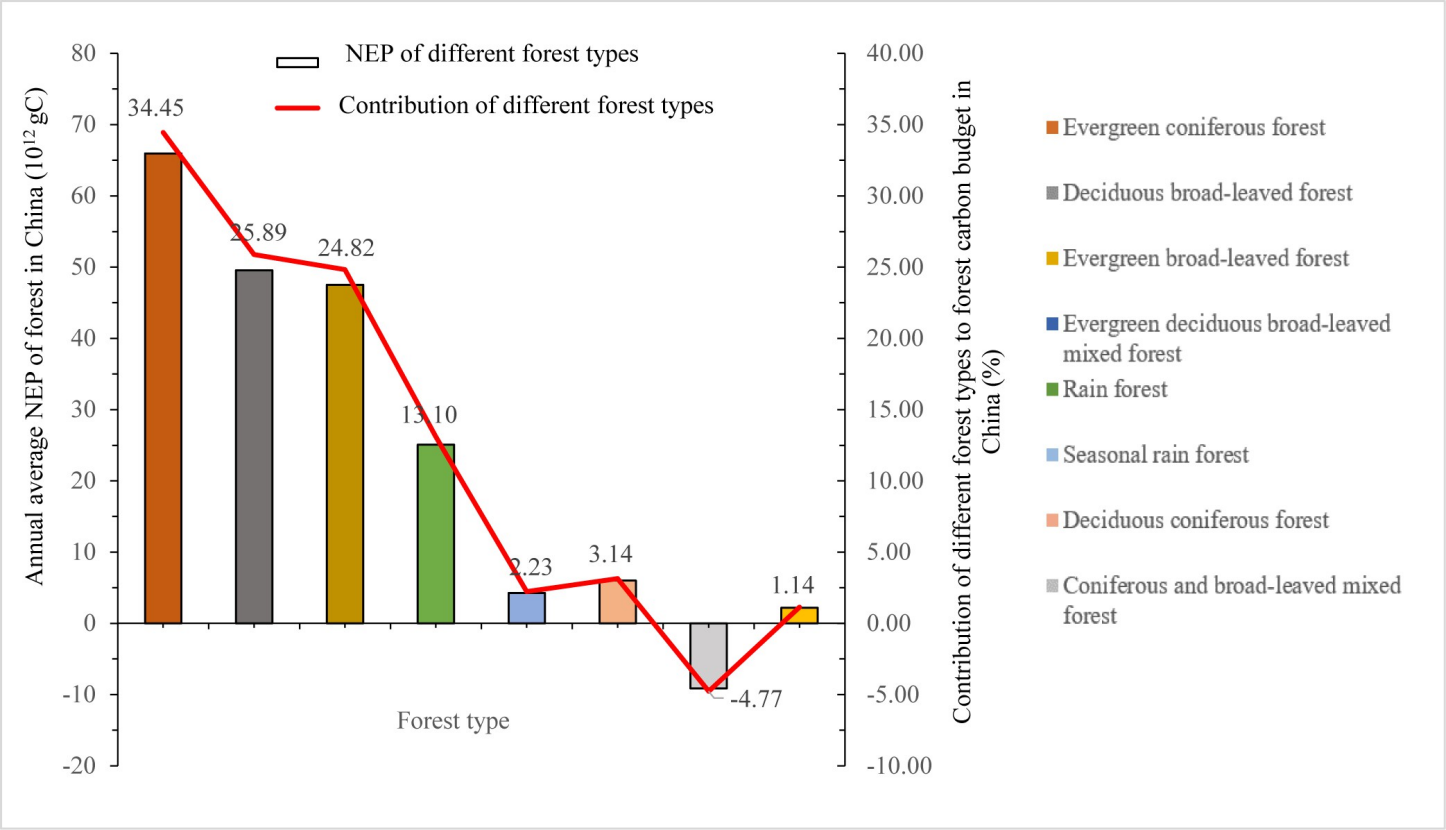
Total NEP of different forest types and their relative contributions to forest carbon budget in China from 2011 to 2020.

Our results showed that the most forest types in China played carbon sink roles. In particular, from 2011 to 2020, the total NEP of ECF forests, DBF forests, CBF forests, EDBF forests, RF forests, SRF forests and CBF forests were 65.95 TgC (1Tg = 10^12^g), 49.56 TgC, 47.52 TgC, 25.08 TgC, 4.26 TgC, 6.01 TgC and 2.19 TgC, respectively. On the contrary, only the DCF forests were carbon sources, and the total amount of carbon released to the atmosphere during 2011–2020 was 9.13 TgC (Fig 5). In addition, the relative contributions of NEP in different forest types to all forests in China were different. From 2011 to 2020, the ECF forests contributed the most NEP (34.45%), followed by DBF forests (25.89%), EBF forests (24.82%), EDBF forests (13.10%), RF forests (2.23%), SRF forests (3.14%) and CBF forests (1.14%).

## Discussion

### Carbon budget and its regional difference of Chinese forests

NEP represents the net absorbed carbon by ecosystems from atmosphere, in other words, carbon budget. NEP is vegetation biomass growth subtracting vegetation autotrophic respiration and soil heterotrophic respiration [29]. In other words, NEP indicates the carbon accumulation rate. The forests are the main body of the terrestrial ecosystem, storing about 45% organic carbon of the terrestrial ecosystem [10], which are characterized by high carbon sink capacity. Cui et al. [30] showed that the forests’ NEP was relatively high in tropical region with good climatic conditions. They also found that forests within same biome also had diverse NEP rates in different regions, which were caused by climate, site location, soil, and so on [31]. By analyzing the simulation results, we found there were distinct spatial changes in NEP of Chinese forests, with a decreasing NEP from Southeast China to Northwest China. Our simulated forests’ NEP with the value of mostly 100 gC·m^2^·a^-1^ ~200 gC·m^2^·a^-1^ in the northeastern China, was basically consistent with the published measurement value of 169 gC·m^2^·a^-1^~187 gC·m^2^·a^-1^ by eddy covariance in the Changbai Mountains [32]. Our simulated NEP of forests in Sichuan Basin of Southwest China, mostly 200 gC·m^2^·a^-1^~300 gC·m^2^·a^-1^, was basically consistent with the measurement NEP of 279 gC·m^2^·a^-1^ in Southwest China [33].

### Carbon budget of different forests

NEP contributions of different forest types to all forests in China were found to vary greatly in this study. High spatial heterogeneities existed in NEP of different forests. The highest NEP was found in the ECF forests which tended to provide carbon sinks all year round. There are several main reasons for this difference: climate, forest structure, vegetation types, phenology, succession stages and environment condition. First, the climate variables are the important factors dominating the NEP variabilities from regional scale to global scale. A study showed that the increased temperature in winter or early spring was conducive to the early maturity and assimilation of the forest, finally affecting the NEP [6]. Second, the forest structures are critical factors determining NEP of forest ecosystems, because different forest types have different age compositions. Tilman et al. [34] demonstrated that younger forests usually had higher net photosynthesis, faster growth and stronger carbon absorption potential than old forests. At the same time, human disturbance made forests young and in early vegetation succession. Thirdly, different forest types have different distribution patterns. Corresponding, their carbon sequestration capacities vary greatly. The evergreen forests have longer growth season and grow in poorer soil, compared with deciduous forest, and the deciduous broad-leaved forests are mainly in 30°N–50°N temperate regions [19]. Jolly et al. [35] pointed out the phenological status significantly affected carbon exchange and NEP of forests, especially in some high latitudes with temperature seasonality and limited photoperiod. Finally, other environmental factors on landscape or regional scales, for instance, geology, topography, surface slope, might important in dominating the variabilities in forests’ NEP, especially in mountainous regions. This is another possible reason for regional difference in NEP.

### Limitations and implication

Some caveats remain that should be examined and paid attention to in future researches when assessing NEP of Chinese forests. As for spatial data, the interpolation accuracies and the spatial resolutions of soil data and meteorological data in China should be constantly increased and improved. In this study, the meteorological station data and soil data were interpolated into grid data (10 km×10 km). On a provincial scale or smaller regional scale, the spatial resolutions of data were needed to be further improved. In addition, the forest succession was not taken into account in this simulation. The next step was to add corresponding modules and enhance the application ability of FORCCHN model in quantitative assessment on the impact of forest succession on NEP of Chinese forests. The used remote sense image data were also led to certain uncertainties in the results. Now, the remote sensing inversion techniques are widely applied for vegetation parameter, making up for ground observation deficiencies [20]. Nevertheless, remote sensing can indirectly monitor the processes of carbon cycle. There are still certain uncertainties of vegetation parameters between retrieved values and indirectly simulation value [36].

According to the statistics from FAO and UNEP, more than half of the global forests were distributed in the Russian, Brazil, Canada, America and China, and the forests in China covered 5% of the land surface in the world [7]. Regrettably, in the second half of the 20th century, human activities severely damaged China’s forests, with an average deforestation of 3.02% every year [37]. In the past few decades, China has implemented many ecological projects and protection laws of forests [38], the forests in China have high carbon sequestration rates and great carbon sink growth potentials, and thus play more and more important roles in emission reduction and regional environment improvement. Nevertheless, at present, the regional differences of forests’ NEP are significant. As the ecological security barrier and important position for developing carbon sink in China, Northwest China still has relatively small NEP values compared with other regions, and even the forests in some forest areas in Northwest China play carbon source roles. In short, in order to slow down climate warming, it is urgent to enhance forest carbon sink through better forest tending and scientific management, for improving the carbon absorption capacity in some forest areas of Northwest China.

## Conclusions

We made use of the FORCCHN model and remote sensing observation data to appraise the NEPs of different forest types and their relative contributions to all forests in China from 1981 to 2020. Several conclusions were drawn as follows. The FORCCHN model had good ability of simulating the NEP of Chinese forests at spatiotemporal scales. Chinese forests were mainly carbon sinks over the past 40 years. The spatial distribution of forests’ NEP demonstrated obvious regional differences, gradually decreasing from Southeast China to Northwest China. It was important to note that the highest values of forests’ NEP from 2011 to 2020 appeared in Southwest China. The values of forests’ NEP in Northeast China and Southeast China were higher. However, the lower NEPs occurred in Northwest China. Relative contributions of NEP in different forest types to all forests in China varied greatly. From 2011 to 2020, the ECF forests contributed the most NEP (34.45%), followed by DBF forests (25.89%), EBF forests (24.82%), EDBF forests (13.10%), RF forests (2.23%), SRF forests (3.14%) and CBF forests (1.14%). Nevertheless, only the DCF forests were considered to be carbon sources, and their released total carbon to the atmosphere from 2011 to 2020 was 9.13 TgC on the whole.

Our research results can provide important information for supporting sustainable development and realizing carbon neutralization target of China, and also offer scientific basis for climate change research. Our results exhibit that carbon absorbed by different forest types in China has played key roles in emission reduction and regional environment improvement over the past several decades. Future work should focus on the accurate dynamic process and mechanism of carbon fixation of different forest types. Consequently, some comprehensive researches should be further conducted through multidisciplinary theories and practices combined multiple methods at the same time, such as modeling and field research.

## Supporting information

S1 Fig Average distribution of NEP of forests in China from 2011 to 2020 (gC·m^-2^·a^-1^). (JPG)

S1 Data The data used for comparison between NEP simulated by FORCECHN model and other research results on the countrywide scale. (XLSX)

S2 Data The data used for comparison of monthly variations between simulated NEP with observed NEE in four ecosystem flux stations in 2003. (XLSX)

S3 Data The data used for total NEP of different forest types and their relative contributions to forest carbon budget in China from 2011 to 2020. (XLSX)

S1 File (DOCX)
