# Supplementary figures and images for: Carbon budget of different forests in China estimated by an individual-based model and remote sensing
Source: PLoS One. 2023 Oct 9;18(10):e0285790. doi: 10.1371/journal.pone.0285790 (PMC10561855; doi:10.1371/journal.pone.0285790)

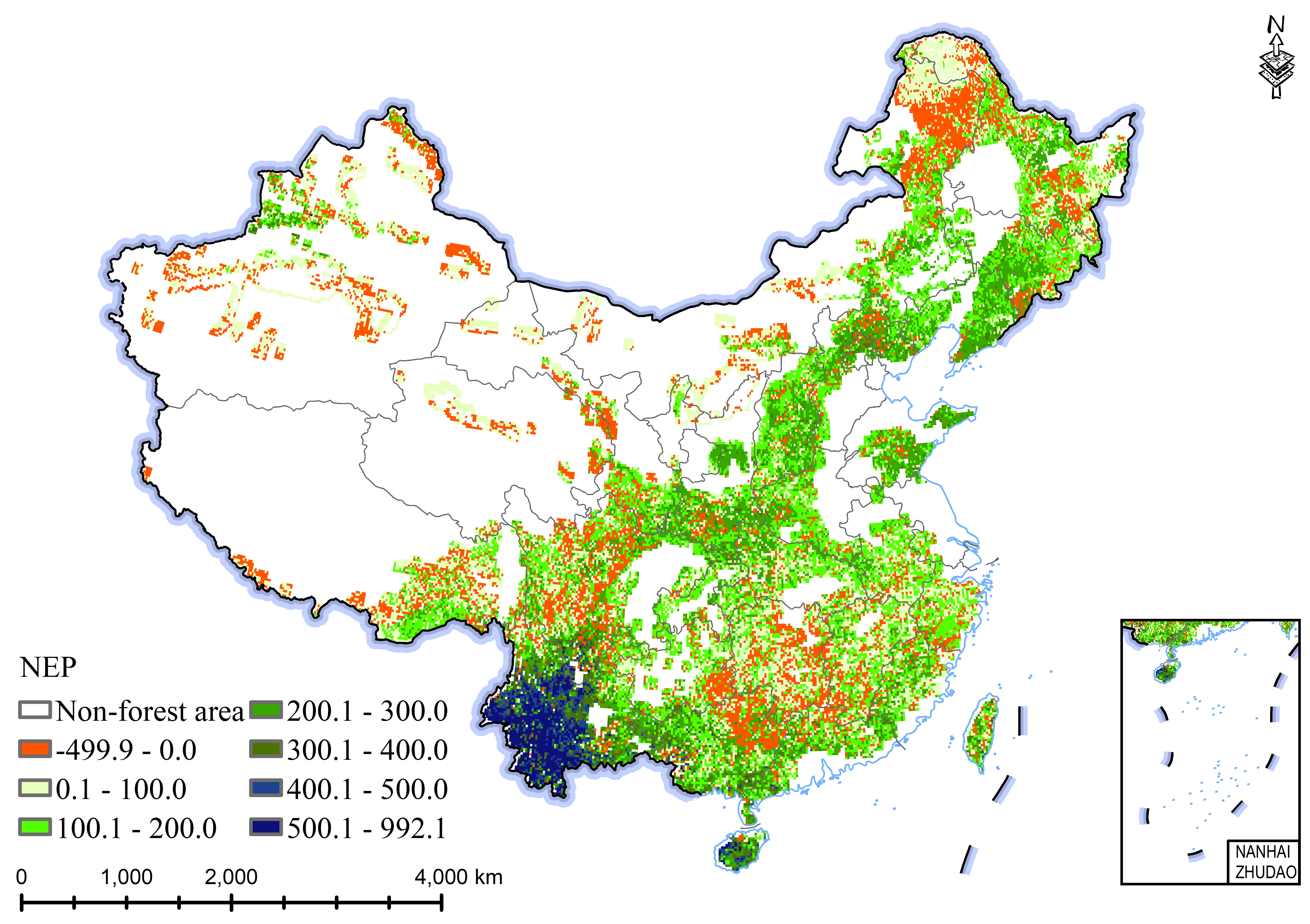

Supplement: S1 Fig — (JPG) [file pone.0285790.s001.jpg]
