# Supplementary material for: Carbon budget of different forests in China estimated by an individual-based model and remote sensing
Source: PLoS One. 2023 Oct 9;18(10):e0285790. doi: 10.1371/journal.pone.0285790 (PMC10561855; doi:10.1371/journal.pone.0285790)
Supplement: S1 File — (DOCX) [file pone.0285790.s005.docx]

Re-sampling

An improved individual-based forest ecosystem carbon budget model FORCCHN (10km×10km)

NDVI data set of AVHRR on NOAA satellite

1:14 million soil quality map from Chinese Academy of Sciences

Daily meteorological data from National Meteorological Information Centre in China

Chinese forest characteristic data

Chinese soil parameters

Daily meteorological datasets

Verification

Gross primary productivity (GPP)

Net primary productivity (NPP)

Ecosystem respiration (ER)

Net ecosystem productivity (NEP)

Carbon in soil and litter

Carbon storages of different forest types and their relative contributions in China

Inter-annual variability in NEP of Chinese forests

Spatio-temporal dynamics of NEP of Chinese forests

Spatio-temporal dynamics of NEP of different forest types in China

Effect of climate change on NEP of different forest types in China
